# Impact of Vegetation Type on Taxonomic and Functional Composition of Soil Microbial Communities in the Northeastern Qinghai–Tibet Plateau

**DOI:** 10.3390/microorganisms13092075

**Published:** 2025-09-06

**Authors:** Xunxun Qiu, Guangchao Cao, Guangzhao Han, Qinglin Zhao, Shengkui Cao, Shuang Ji

**Affiliations:** 1 College of Geographical Sciences, Qinghai Normal University, Xining 810008, China; 202247341007@stu.qhnu.edu.cn (X.Q.);; 2 Qinghai Provincial Key Laboratory of Physical Geography and Environmental Processes, Xining 810008, China; 3 Graduate School, Qinghai Normal University, Xining 810008, China

**Keywords:** alpine region, different vegetation types, soil microbial community structure, influence factor, function prediction, Qinghai-Tibet Plateau

## Abstract

Soil microbial communities are pivotal in maintaining ecosystem functions, particularly in alpine regions with highly heterogeneous environmental conditions. However, the influence of vegetation type on soil microbial communities in high-elevation areas remains insufficiently understood. This study investigates the dynamics of soil microbial communities across grassland, shrubland, and forest ecosystems on the southern slope of the Qilian Mountains. Soil bacterial and fungal communities were examined using high-throughput 16S rRNA and ITS gene sequencing, and their potential ecological functions were inferred using the FAPROTAX and FUNGuild databases. Analysis of similarity (ANOSIM) based on Bray–Curtis distances revealed significant differences in bacterial and fungal community structures among vegetation types, with forest soils showing greater intra-group variability and more distinct microbial assemblages. Acidobacteriota and Proteobacteria were the dominant bacterial phyla, while Basidiomycota and Ascomycota predominated among fungi. Fungal communities in forest soils were dominated by ectomycorrhizal taxa, closely linked to coniferous forests dominated by *Picea crassifolia*. Overall, the structure and functional diversity of soil microbial communities were governed by soil physicochemical properties, particularly soil pH, which emerged as a key influencing factor. These findings deepen our understanding of microbial ecological processes in alpine environments and offer valuable insights for effective vegetation management and ecosystem conservation in mountainous regions.

## 1. Introduction

Soil microorganisms are fundamental in maintaining the connections between aboveground and belowground components of ecosystems and act as critical drivers of plant biodiversity and ecosystem productivity. They participate in nearly all soil biogeochemical processes, undertaking essential ecological roles such as facilitating nutrient cycling, optimizing plant mineral uptake, and mitigating nutrient limitations across diverse environments including forests, grasslands, shrublands, and agricultural systems [1,2,3]. Accumulating evidence indicates that variations in vegetation types significantly reshape the composition and functional attributes of soil microbial communities [4,5,6]. Distinct vegetation types contribute unique forms of litter and root-derived exudates, thereby influencing both the availability and the chemical profile of soil nutrients [7,8,9]. Therefore, studying the spatial distribution and adaptive responses of soil microbial communities under different vegetation types is crucial for enhancing our understanding of biogeochemical cycling processes and the mechanisms regulating ecosystem function.

Plant–soil interactions exert strong selective pressures on microbial community composition, abundance, and spatial distribution, thereby directly and indirectly influencing plant growth and shaping plant community composition and diversity [10]. Soil microorganisms are widely recognized as early indicators of ecosystem changes [11]. Under different vegetation types, shifts in soil moisture and texture influence the release of root exudates [12], regulate the diffusion of nutrients and metabolites, and ultimately shape soil properties and microbial community structures [13,14,15]. For example, Sun et al. [16] observed that elevational variations in vegetation and soil characteristics significantly influenced alpine soil bacterial communities in Mount Gongga, China. Similarly, Hernández-Cáceres et al. [17] demonstrated that vegetation-mediated microenvironments modulated microbial activity and functional diversity in the French Alps, with soil physicochemical parameters being the primary drivers. Bastida et al. [18] highlighted that soil carbon stocks are closely associated with the microbial diversity-to-biomass ratio, while Jiao et al. [19] found that microbial community composition, rather than diversity alone, better predicts multi-nutrient cycling efficiency. Therefore, vegetation–microbe interactions have profound ecological significance, and understanding spatial variations in microbial community composition across vegetation types has become a central topic in microbial ecology. Identifying the major environmental drivers underlying these patterns is essential [20]. Although considerable progress has been made in various ecosystems, systematic investigations into how vegetation types influence soil properties and microbial community structures under alpine conditions, where plant growth is constrained by low temperatures and limited precipitation, remain scarce. Addressing this gap is crucial for advancing our understanding of microbial ecology in high-elevation environments and informing biodiversity conservation strategies [21].

The Qinghai–Tibet Plateau exhibits extreme environmental characteristics, including high elevations, intense solar radiation, low atmospheric oxygen concentrations, large diurnal temperature fluctuations, and highly heterogeneous hydrothermal regimes [22]. It is widely acknowledged as one of the world’s foremost biodiversity hotspots [23]. Situated in the northeastern sector of the Plateau, the Qilian Mountains act as a vital ecological transition zone within the arid landscapes of northwestern China. This region is distinguished by substantial altitudinal variation, complex topographical features, and pronounced climatic heterogeneity, thereby supporting a representative alpine ecosystem by distinct vegetation zonation and exceptional biodiversity. These unique environmental and ecological conditions render the Qilian Mountains an ideal natural laboratory for investigating vegetation distribution patterns in alpine ecosystems [24,25]. Against this backdrop, the present study focused on the southern slope of the Qilian Mountains, examining three representative vegetation types—grassland, shrubland, and forest—across an elevational gradient ranging from 2600 to 4100 m. This study aimed to assess whether significant differences exist in soil bacterial and fungal diversity among distinct vegetation types within the study area and to evaluate the influence of vegetation type on the composition and functional profile of soil microbial communities and to identify the primary environmental factors driving this influence.

## 2. Materials and Methods

### 2.1. Study Area

The southern slope of the Qilian Mountains, situated in Qinghai Province in the northeastern sector of the Qinghai–Tibet Plateau, was selected as the study area. Geographically, it extends from 98°08′13″ E to 102°38′16″ E and from 37°03′17″ N to 39°05′56″ N, encompassing approximately 24,000 km^2^. Elevations range between 2286 and 5210 m, with a mean altitude of 3800 m. The region receives between 2200 and 2900 h of sunshine annually and maintains a low mean annual temperature of approximately −5.9 °C. The plant growing season coincides with the period of elevated temperature and precipitation, with the majority of rainfall occurring from June to August, and an average annual precipitation ranging from 300 to 400 mm. Characterized by long, cold winters and brief, cool summers, the area represents a typical alpine ecosystem. The landscape is predominantly mountainous with considerable variation in elevation. The interaction of topographic complexity and climatic variability leads to pronounced vertical vegetation zonation, primarily comprising grasslands, shrublands, and forests [26]. The forest ecosystem is largely dominated by cold-temperate coniferous species, especially *Picea crassifolia*, often intermixed with *Sabina przewalskii*. Common broadleaf species include *Populus davidiana*, *Betula platyphylla*, and other birch species. Shrublands are primarily constituted by *Potentilla fruticosa*, various *Salix* species, and *Caragana jubata*. The herbaceous layer is dominated by plants from the Poaceae, Artemisia, and Carex genera [27]. Major soil types present within the study region include mountain forest soil, grey-cinnamon soil, calcareous chestnut soil, black calcareous soil, alpine meadow soil, meadow soil, and alpine desert soil [28].

### 2.2. Sampling and Sample Treatment

In 2022, vegetation and soil sampling was carried out across three representative ecosystem types within the study area. Forest plots were selected from coniferous stands dominated by *P. crassifolia* and *S. przewalskii*, shrubland plots consisted of mixed shrub communities primarily composed of *P. fruticosa* and *Potentilla glabra*, and grassland plots encompassed alpine meadows and alpine steppes. Sampling sites were distributed along an elevational gradient ranging from 2600 to 4100 m, with 200 m vertical intervals between sampling bands. At each elevation, three replicate plots were established for each vegetation type (forest, shrubland, and grassland), ensuring comparable environmental conditions (e.g., slope, minimal anthropogenic disturbance). Plot sizes were designated as 10 m × 10 m for forest, 5 m × 5 m for shrubland, and 1 m × 1 m for grassland. All replicate plots were spaced at least 10 m apart. A total of 72 plots were established across eight elevational bands. The 0–20 cm soil depth was selected because it has relatively high root density and biological activity, both of which significantly influence microbial community composition and functional dynamics in terrestrial ecosystems.

Mature leaves from trees, shrubs, and herbaceous plants were collected at each sampling site, resulting in 72 composite leaf samples, with 24 samples representing each vegetation type. These samples were used to measure the carbon and nitrogen content of the plant leaves. Furthermore, 24 composite stem samples were obtained from trees and shrubs, resulting in a total of 48 stem samples. Fine roots were extracted from the 0–20 cm soil layer using a hot-water-assisted sieving method [29], yielding 72 fine root samples. Fresh plant materials were immediately transported to the laboratory, washed thoroughly with deionized water, enzyme-deactivated by steaming at 105 °C for 30 min, and subsequently oven-dried at 65 °C for later analysis. Soil samples were obtained using a 5 cm diameter auger, with three random subsamples collected from each plot following an S-shaped sampling pattern. These subsamples were combined to form one composite soil sample per plot, producing a total of 72 composite soil samples. Large stones and undecomposed root debris were manually removed. Portions of fresh soil were stored at 0–4 °C for immediate physicochemical analysis, while the remaining soils were air-dried. For bulk density measurements, additional soil cores were collected using a ring knife from the 0–20 cm layer, yielding 72 samples across all vegetation types. All sampling tools, including augers, sieves, and centrifuge tubes, were sterilised prior to use following microbial sampling protocols. For microbial community analysis, one composite soil sample per vegetation type was generated at each elevation by mixing soils from the three replicate plots. These microbial samples were immediately placed in sterile centrifuge tubes and preserved in liquid nitrogen at −80 °C. A total of 24 soil samples were collected for subsequent analysis of community structure and diversity.

### 2.3. Analysis of Plant Characteristics and Soil Physicochemical Properties

Plant samples, including roots, stems, and leaves of trees and shrubs, as well as roots and leaves of herbaceous plants, were ground into fine powder prior to analysis. The carbon content was determined using the external heating method with potassium dichromate, while nitrogen content was measured with an AA3 continuous flow analyzer. Soil water content (SWC) was assessed by using the oven-drying method, and soil bulk density (BD) was determined using the ring-knife method. Soil texture (mechanical composition) was analyzed with a Mastersizer 2000 laser particle size analyzer (Malvern Panalytical, Malvern, UK). Electrical conductivity (EC) and pH were measured using a conductivity meter and a pH meter, respectively. Soil organic carbon (SOC) and total nitrogen (TN) were quantified with an elemental analyzer. Total phosphorus (TP) and available phosphorus (AP) concentrations were assessed using the antimony method. Total potassium (TK) and available potassium (AK) were measured through NaOH fusion followed by flame photometry. Alkali-hydrolysable nitrogen (AN) was determined using the alkali diffusion method. Microbial biomass carbon (MBC), nitrogen (MBN), and phosphorus (MBP) were evaluated using the chloroform fumigation extraction method. All procedures followed the standard protocols outlined in Soil Agrochemical Analysis [30]. Soil particle size classification was based on the international system proposed by Su et al. [31].

### 2.4. Soil DNA Extraction, PCR Amplification, and Illumina Sequencing

Genomic DNA was extracted from soil samples using the E.Z.N.A.^®^ Soil DNA Kit (Omega Bio-tek, Norcross, GA, USA) according to the manufacturer’s instructions. DNA integrity was verified by 1% agarose gel electrophoresis, and concentration and purity were determined using a NanoDrop 2000 spectrophotometer (Thermo Scientific, Waltham, MA, USA). The V3V4 region of the bacterial 16S rRNA gene was amplified using primers 338F (5′-ACTCCTACGGGAGGCAGCAG-3′) and 806R (5′-GGACTACHVGGGTWTCTAAT-3′), each containing a unique barcode sequence [32]. For fungal community analysis, the ITS2 region was amplified using the primer pair gITS7 (5′-GTGARTCATCGARTCTTTG-3′) and ITS4 (5′-TCCTCCGCTTATTGATATGC-3′). PCR products were recovered by 2% agarose gel electrophoresis and subsequently purified with a PCR Clean-Up Kit (YuHua Biotech, Shanghai, China). The PCR reaction mixture consisted of the following components: 4 μL of 5× TransStart FastPfu buffer, 2 μL of 2.5 mM dNTPs, 0.8 μL of forward primer (5 μM), 0.8 μL of reverse primer (5 μM), 0.4 μL of TransStart FastPfu DNA polymerase, and 10 ng of template DNA, with the final volume adjusted to 20 μL using nuclease-free water. The amplification program was set as follows: initial denaturation at 95 °C for 3 min, followed by 30 cycles of denaturation at 95 °C for 30 s, annealing at 55 °C for 30 s, and extension at 72 °C for 45 s, with a final extension at 72 °C for 10 min. The PCR products were recovered using 2% agarose gel electrophoresis, purified with a PCR Clean-Up Kit, and quantified using a Qubit 4.0 fluorometer (Thermo Fisher Scientific, USA). Sequencing libraries were constructed using the NEXTFLEX Rapid DNA-Seq Kit. Raw sequencing data were quality-controlled using fastp software (version 0.19.6), and paired-end reads were merged using FLASH software (version 1.2.11). The merged sequences were clustered into operational taxonomic units (OTUs) at a 97% similarity threshold using UPARSE software (v7.1). Taxonomic annotation of bacterial and fungal OTUs was performed using the RDP Classifier, with bacterial OTUs aligned to the SILVA 16S rRNA gene database (v138) and fungal OTUs aligned to the UNITE ITS database (v8.0). Community composition was then analyzed at different taxonomic levels for each sample, and all data were normalized prior to further analysis. High-throughput sequencing was conducted on the Illumina PE300/PE250 platform by Shanghai Majorbio Bio-Pharm Technology Co., Ltd. (Shanghai, China).

### 2.5. Statistical Analyses

Alpha diversity indices, including Chao1 and Shannon, were calculated using the Mothur software package [33] (http://www.mothur.org/wiki/Calculators, accessed on 20 March 2025). Analysis of variance (ANOVA) was performed using IBM SPSS Statistics (version 26, IBM Corp., Armonk, NY, USA), and Duncan’s multiple range test was used to determine significant differences in soil environmental factors among different vegetation types. Differences in alpha diversity among soil microbial communities from different vegetation types were evaluated with the Wilcoxon rank-sum test. Microbial community composition similarities were assessed based on Bray–Curtis distances, and community structural differences across groups were tested using permutational multivariate analysis of variance (PERMANOVA). Linear discriminant analysis effect size (LEfSe) was applied to identify bacterial and fungal phyla exhibiting significantly different relative abundances among vegetation types (LDA > 2, *p* < 0.05) [34]. Distance-based redundancy analysis (db-RDA) was used to explore the key factors influencing the composition and diversity of soil microbial communities. Subsequently, linear regression models were used to examine the relationships between key environmental variables identified in db-RDA and alpha diversity indices [35].

## 3. Results

### 3.1. Plant and Soil Characteristics Under Different Vegetation Types

The vegetation traits, along with the physicochemical and biological attributes of soils across the grassland, shrubland, and forest ecosystems in the study area, are summarized in Table 1. Significant variations in carbon and nitrogen concentrations were detected in the roots and leaves of plants among the three vegetation types. Forest vegetation showed the highest carbon contents in both roots and leaves, which were significantly greater than those observed in shrubland and grassland (*p* < 0.05). Root nitrogen content was the highest in shrubland soils, significantly surpassing that of grasslands (*p* < 0.05), whereas leaf nitrogen content was highest in grasslands, significantly exceeding that in forest ecosystems (*p* < 0.05). The forest soils exhibited the greatest SOC levels, significantly higher than those found in grassland soils (*p* < 0.05). Soils under grassland and shrubland were weakly alkaline, whereas forest soils were weakly acidic. Furthermore, microbial biomass nitrogen (MBN) content was the highest in forest soils and significantly greater than the microbial available nitrogen (MAN) content measured in grassland soils (*p* < 0.05).

### 3.2. Soil Microbial Diversity and Community Composition Under Different Vegetation Types

**Figure 1:**
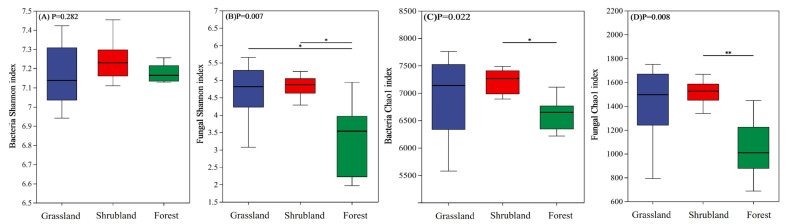
α diversity of bacterial and fungal communities in different vegetation types. Shannon index (**A**) and Chao l index (**C**) for bacteria, Shannon index (**B**) and Chao l index (**D**) for fungi. *, *p* < 0.05; **, *p* < 0.01. Group statistical significance was assessed by one-way ANOVA followed by Tukey’s HSD test.

The alpha diversity metrics for bacterial and fungal communities across the different vegetation types are illustrated in Figure 1. According to the Shannon index, no significant variation in bacterial diversity was detected among grassland, shrubland, and forest soils (Figure 1A). However, fungal diversity was markedly higher in grassland and shrubland soils compared to forest soils (Figure 1B). Based on the Chao1 index, both bacterial and fungal richness were significantly elevated in shrubland soils relative to forest soils, whereas fungal richness did not differ significantly between shrubland and grassland soils (Figure 1C,D).

**Figure 2:**
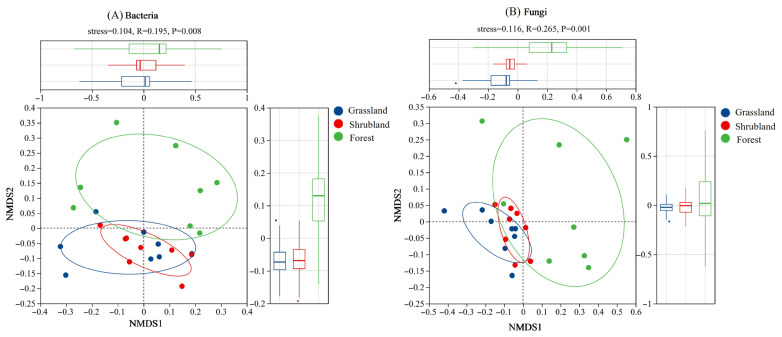
Nonmetric multidimensional scale (NMDS) ranking maps based on Bray–Curtis distances for bacterial communities (**A**) and fungal communities (**B**) of different vegetation types. The upper and right-side boxplots represent the median, interquartile range, and potential outliers for the different groups along the NMDS1 and NMDS2 axes, respectively.

ANOSIM analysis using Bray–Curtis distances indicated significant differences in bacterial (Figure 2A) and fungal (Figure 2B) community structures among the three vegetation types (*p* < 0.01). Compared to grassland and shrubland soils, the microbial communities in forest soils exhibited greater variability. In contrast, microbial communities in grassland and shrubland soils were more tightly clustered, reflecting greater internal similarity and indicating more stable community structures.

**Figure 3:**
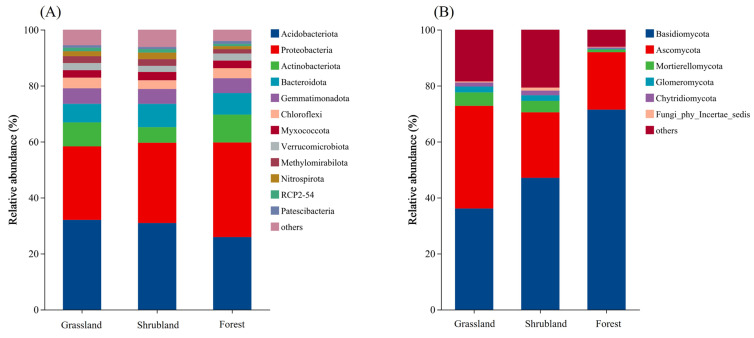
Composition of soil bacterial (**A**) and fungal (**B**) communities in different vegetation types based on phylum relative abundance.

Across the soils from the three vegetation types, bacterial phyla with a relative abundance > 1% included *Acidobacteriota*, *Proteobacteria*, *Actinobacteriota*, *Bacteroidota*, *Gemmatimonadota*, *Chloroflexi*, *Myxococcota*, *Verrucomicrobiota*, *Methylomirabilota*, *Nitrospirota*, RCP2-54, and *Patescibacteria* (Figure 3A). Among these, *Acidobacteriota* and *Proteobacteria* were dominant, accounting for 58% in grassland soils and approximately 60% in both shrubland and forest soils. Fungal phyla with a relative abundance > 1% included *Basidiomycota*, *Ascomycota*, Mortierellomycota, *Glomeromycota*, *Chytridiomycota*, and several unclassified phyla (incertae sedis) (Figure 3B). *Basidiomycota* and *Ascomycota* together constituted 72.84% of fungal communities in grassland soils, 70.51% in shrubland soils, and 91.99% in forest soils. Notably, fungal phyla with relative abundances below 1% accounted for 18.45% and 20.7% in grassland and shrubland soils, respectively, suggesting greater diversity of rare or poorly classified fungal phyla in these ecosystems compared to forests.

**Figure 4:**
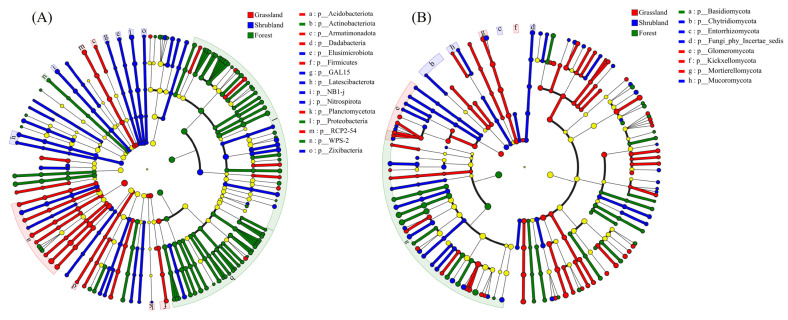
LEfSe of soil bacterial (**A**) and fungal (**B**) communities. Nodes in different colors indicate significant enrichment in different vegetation types, while light yellow nodes represent no significant difference among vegetation types.

LEfSe analysis identified significant differences in the relative abundances of microbial taxa among vegetation types. A total of 15 bacterial phyla (Figure 4A) and eight fungal phyla (Figure 4B) exhibited significant enrichment patterns (*p* < 0.05). In grassland soils, bacterial phyla such as Acidobacteriota, Armatimonadota, Dadabacteria, Firmicutes, Planctomycetota, and RCP2-54 were significantly enriched, along with the fungal phyla Glomeromycota, Kickxellomycota, and Mortierellomycota. In shrubland soils, Elusimicrobiota, GAL15, Latescibacterota, NB1-j, Nitrospirota, and Zixibacteria dominated the bacterial community, while Chytridiomycota, Entorrhizomycota, and an unclassified fungal group (incertae sedis) were predominant among fungi. Forest soils were characterised by enriched bacterial phyla such as Actinobacteriota, Proteobacteria, and WPS-2, and a fungal community dominated by Basidiomycota.

### 3.3. Prediction of Functional Groups in Soil Bacterial and Fungal Communities

**Figure 5:**
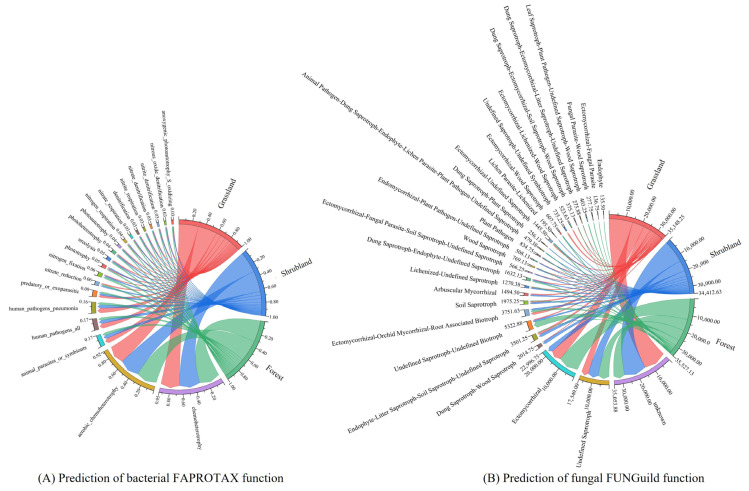
Predicted functional groups in soil bacterial communities based on FAPROTAX (**A**) and in soil fungal communities based on FUNGuild (**B**) of different vegetation types.

Based on taxonomic annotations of 16S and ITS sequences and functional predictions using the FAPROTAX and FUNGuild databases, a total of 61 bacterial and 97 fungal functional groups were identified across the grassland, shrubland, and forest ecosystems. The 20 most abundant functional groups for each vegetation type were selected and visualised through chord diagrams to display the functional profiles of the bacterial (Figure 5A) and fungal (Figure 5B) communities. Overall, bacterial functional composition exhibited only minor variation among vegetation types, with the majority of functions related to carbon cycling. Additionally, several functional groups were linked to animal-associated pathogenicity, such as animal parasites, human pathogens, and pneumonia-associated microbes, along with nitrogen cycling processes like nitrate reduction and nitrogen fixation. Forest soils exhibited the highest proportions of genes associated with both carbon cycling (66.38%)—including chemoheterotrophy, aerobic chemoheterotrophy, and photoheterotrophy—and nitrogen cycling (9.73%), encompassing nitrate reduction, nitrogen fixation, denitrification, nitrite respiration, nitrate denitrification, and nitrite denitrification. These percentages exceeded those observed in grassland (65.02% and 7.63%, respectively) and shrubland soils (59.62% and 6.21%, respectively). In contrast, shrubland soils showed the highest relative abundance (26%) of functional groups associated with animal parasitism and pathogenicity, including animal parasites, human pathogens, pneumonia-related pathogens, and predator or exoparasitic groups, markedly higher than corresponding proportions in grassland (19%) and forest (14%) soils.

A substantial proportion of fungal functional groups were classified as unknown, followed by undefined saprotrophs. Ectomycorrhizal fungi, vital for nutrient cycling, form symbiotic relationships with plants to facilitate nutrient exchange. The distribution of dominant fungal functions varied significantly among vegetation types. Unknown functional groups accounted for the largest proportion in shrubland (40.97%) and grassland (40.06%) soils, greatly exceeding that observed in forest soils (19.33%). Conversely, ectomycorrhizal fungi were far more abundant in forest soils (35.77%) compared to shrubland (20.14%) and grassland (9.33%) soils.

### 3.4. Environmental Drivers of Soil Microbial Community Differences Across Vegetation Types

**Figure 6:**
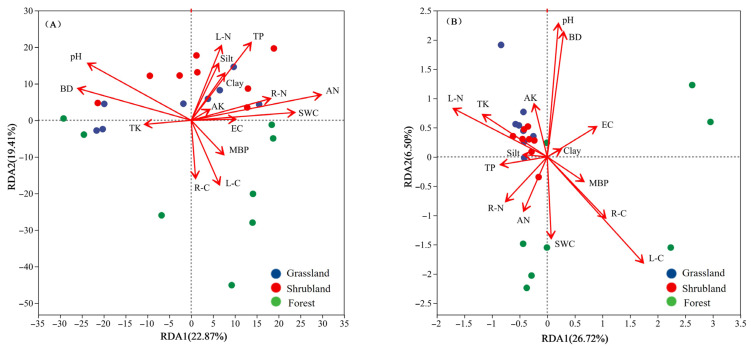
Relationship of composition of bacterial (**A**) and fungal (**B**) communities with vegetation characteristics, and soil physicochemical properties.

To enhance the robustness of the correlation analysis between environmental factors and microbial community structures, a variance inflation factor (VIF) analysis was performed on 21 environmental variables (Supplementary Table S1) to eliminate multicollinearity. Variables with VIF values exceeding 20—including soil sand content, SOC, MBC, and MBN—were excluded before conducting RDA. As presented in Figure 6, the RDA results accounted for 42.28% and 33.22% of the variation in bacterial and fungal phyla, respectively, influenced by the environmental variables across the different vegetation types. Overall, soil pH, BD, AN, TP, and L-C were identified as the primary environmental factors shaping bacterial community structure and diversity (*p* < 0.05). In grassland soils, the composition of bacterial communities is primarily determined by soil bulk density (BD) and total phosphorus (TP). In shrubland soils, soil pH serves as the predominant driver, whereas in forest soils, litter carbon (L-C) plays the key role. For fungal communities, significant predictors (*p* < 0.05) include soil pH, BD, L-C, litter nitrogen (L-N), and soil water content (SWC). Specifically, fungal communities in grassland and shrubland soils show the strongest associations with soil pH and L-N, while those in forest soils are more closely linked to SWC.

## 4. Discussion

### 4.1. Effect of Vegetation Type on Taxonomic Composition of Microbial Communities

Plants actively shape the composition and diversity of soil microbial communities through selective interactions within the rhizosphere, creating a more favorable microhabitat for their own development [36]. Fungal diversity was higher in grassland and shrubland soils than in forest soils, and bacterial and fungal richness was greater in shrubland soils. Forest microbial communities showed high within-group variability and clear between-group differences among vegetation types. These patterns suggest that vegetation type, via its effects on soil physicochemical properties, underlies the observed variation in microbial diversity and community composition. During plant growth, the simultaneous uptake of soil nutrients and release of a wide spectrum of root exudates influence soil conditions, affecting microbial diversity [4,37]. Higher plant diversity leads to more heterogeneous exudates and litter inputs, thus promoting a greater diversity and richness of soil microorganisms [38]. The forests on the southern slopes of the Qilian Mountains are dominated by Qinghai spruce, accompanied by scattered individuals of Qilian juniper, forming patchy, middle-aged, single-species coniferous stands, mainly distributed on shady slopes [39]. This simplified community structure likely accounts for the lower microbial richness observed, consistent with the previous findings that coniferous forests, due to their chemically uniform and hardly decomposing litter, as well as limited understory diversity, offer narrower ecological niches for microbial colonisation [40,41]. Shrub vegetation often creates distinct “fertile islands” in arid regions by concentrating nutrients beneath their canopies [42]. Compared to grasslands, shrub litter is more lignified, offering substrates for fungi specialised in lignin degradation, such as white rot fungi [43,44]. The more chemically diverse root exudates in shrubs further promote specific associations with bacterial and fungal communities. In contrast, although forest ecosystems may contribute higher total litter mass, the high carbon-to-nitrogen (C:N) ratio of coniferous litter, along with the composition of acidic coniferous litter sometimes containing toxic components, could inhibit microbial activity [45], compounded by chemically uniform root exudates from *P. crassifolia*.

An increasing number of studies have confirmed that the overall composition of soil bacterial and fungal phyla across the Qilian Mountains is relatively consistent, with exceptions limited to specialised habitats such as glacier forefields. The dominant bacterial phyla identified in this study—Acidobacteriota, Proteobacteria, Actinobacteriota, Bacteroidota, Gemmatimonadota, Chloroflexi, and Myxococcota—align well with earlier findings [46,47]. Similarly, the main fungal phyla—Basidiomycota, Ascomycota, Mortierellomycota, Glomeromycota, Chytridiomycota, and incertae sedis taxa—are consistent with previous reports [48,49]. Our results indicated no major differences in the taxonomic composition of microbial communities among forest, shrubland, and grassland soils, suggesting high stability of microbial assemblages across vegetation types. However, the results may also indicate with high probability that the phylum-level taxonomic resolution, especially for fungal communities, is not sufficient to capture the differences among communities. This consistency is likely shaped by strong environmental filtering effects exerted by the harsh alpine conditions of the Qilian Mountains, including high elevation, intense ultraviolet radiation, and arid, cold climates. Such stresses permit only well-adapted microbial taxa to survive [50]. Moreover, the general nutrient poverty and near-neutral to weakly alkaline pH of soils in the region selectively favour taxa like Gemmatimonadota and Mortierellomycota, corroborated by our LEfSe analysis results [51]. Although the Qilian Mountains impose geographic isolation and dispersal limitations on microbial taxa, local gradients in precipitation and temperature may still facilitate community convergence at small spatial scales [52].

### 4.2. Effect of Vegetation Type on Functional Composition of Microbial Communities

Taxonomic composition and relative abundance data alone are insufficient for accurately inferring microbial ecological functions. Analyses based on functional genes, particularly those linked to organic matter mineralization, nutrient cycling, and plant–microbe–soil interactions in the rhizosphere, offer a more comprehensive understanding of microbial roles [53]. In this study, microbial functional annotation for bacterial and fungal communities across forest, shrubland, and grassland soils was conducted using 16S and ITS taxonomic data through the FAPROTAX and FUNGuild databases.

The results revealed that carbon cycling, nitrogen cycling, and animal-pathogen-related processes were the dominant functions in bacterial communities across all vegetation types. Forest soils exhibited the highest proportions of genes associated with both carbon and nitrogen cycling, suggesting that microbial communities in forests are functionally more active in these biogeochemical processes than those in shrubland or grassland ecosystems. This observation is consistent with findings by Wang et al. [21] and may be attributed to the higher primary productivity of forest ecosystems. Forest floors, typically covered by lignin- and cellulose-rich litter, create moist, shaded conditions that slow decomposition rates, facilitate humus accumulation, and enhance the availability of carbon and nitrogen sources for soil microbes [54].

In contrast, shrubland soils presented the highest relative abundance of functional groups associated with animal parasitism and pathogenicity. Although soil bacteria themselves are generally not parasitic, soils can act as reservoirs for parasite eggs and larvae linked to animal hosts [55]. Similar ecological patterns have been reported in the northeastern United States [56] and northern Belgium [57], where shrubland habitats demonstrated higher infection rates of *Borrelia burgdorferi* and its vector *Ixodes ricinus* compared to grassland or forested areas. This could be explained by several ecological mechanisms. Shrublands offer moderate microclimatic conditions—more humid than grasslands and more ventilated than forests—that promote parasite and pathogen survival [58]. Additionally, the dense shrub architecture provides abundant shelter for hosts and vectors, enhancing the stability of host–vector–pathogen networks. The ecotonal nature of shrublands also amplifies “edge effects,” increasing cross-species contact and facilitating pathogen transmission [59]. Compared to grasslands, shrublands tend to attract a greater diversity of small mammals, birds, and reptiles, many of which serve as intermediate or definitive hosts, while forests with lower prey density and stronger predator populations may suppress pathogen transmission [60].

Fungal functional groups in shrubland and grassland soils showed a higher proportion of unclassified functions compared to forest soils, where fungi were more functionally resolved. Nevertheless, among the classified fractions, these open ecosystems supported a range of ecological strategies, including endomycorrhizal, ectomycorrhizal, and root endophytic associations, reflecting the functional diversity promoted by herbaceous and shrub vegetation. The higher proportion of unclassified functions suggests that soil fungi in these ecosystems may engage in unique, less-understood interactions, contributing to their ecological complexity. In contrast, the dominance of EcM fungi in forest soils reflects the strong dependence of *P crassifolia*—the main tree species in the study area’s forests—on associations [61,62]. Although plant species richness is generally higher in grassland and shrubland ecosystems [63], the ecological functions of associated fungi, such as arbuscular mycorrhizal fungi (AMF) and endophytes, remain poorly annotated [64]. In contrast, *P. crassifolia* forms specific mutualistic partnerships with fungal genera such as *Russula* and *Cortinarius*, which substantially enhance EcM fungal abundance in forest ecosystems [65].

### 4.3. Environmental Conditions Jointly Regulate Soil Microbial Community Differences Across Vegetation Types

According to the classical theory of microbial community assembly, environmental conditions are considered key factors in shaping soil microbial community structure [66]. In this study, the bacterial community composition in grassland soils is primarily influenced by soil bulk density (BD) and total phosphorus (TP), while that in shrubland soils is mainly influenced by soil pH, and the bacterial community composition in forest soils is more influenced by leaf carbon content (L-C). Soil organic carbon (SOC) was excluded from the RDA due to its strong collinearity with leaf carbon (LC), which could lead to redundancy in the model. Grasslands typically experience higher weathering rates and stronger human–animal disturbances, resulting in compacted soil and nutrient limitations. In such environments, microorganisms must adapt to the challenges of limited space and nutrient availability, making them highly sensitive to soil BD and TP [67]. The pH of shrubland soils in the study area is relatively high, and previous studies have shown that soil pH significantly impacts the availability of nutrient ions and controls microbial enzyme activity, cell membrane function, and community composition [68]. Therefore, microorganisms in shrubland soils are more dependent on pH changes to adapt to the environment. Compared to grasslands and shrublands, forest ecosystems provide abundant litter and root exudates, forming a complex carbon input system. Soil bacteria are highly sensitive to both the type and availability of these carbon sources, and different vegetation types can directly influence bacterial community composition and metabolic functions through variations in substrate composition and quality [48].

For soil fungi, the community composition in grassland and shrubland soils is more influenced by soil pH and leaf nitrogen (L-N), while that in forest soils is more influenced by soil water content (SWC). Many studies have shown that changes in soil pH can lead to shifts in microbial communities, including fungi. Soil pH determines the survival thresholds of microorganisms, directly affects enzyme activity, and indirectly regulates nutrient availability through ion exchange mechanisms [68]. In the study area, soil pH and L-N concentrations in grassland and shrubland ecosystems are significantly higher than in forests, indicating a close relationship between pH and nitrogen concentration, with both factors jointly influencing the distribution patterns of microorganisms in grassland and shrubland ecosystems. In forest ecosystems, soil water content (SWC) emerged as the primary factor influencing fungal community composition. This is likely because, in the studied region, forest soils—particularly under dense coniferous canopies—receive less direct solar radiation and experience slower evaporation, leading to greater temporal and spatial variation in SWC compared to grassland and shrubland soils. Such variation can strongly affect oxygen availability, litter decomposition rates, and nutrient mineralization, all of which are critical for fungal growth [69,70]. In high-altitude forests, periodic water fluctuations may therefore exert a stronger selective pressure on fungal communities than in more open ecosystems, where soils are typically drier and SWC remains consistently low. Moreover, soil moisture regulates thermodynamics and hydrodynamics, promotes nutrient uptake by plants, and indirectly shapes microbial community composition by enriching the soil nutrient pool [71].

Although the precise mechanisms driving plant–microbe interactions remain to be fully clarified, increasing evidence suggests that vegetation types can modify microbial structure and functionality by altering litter input quality, root exudate profiles, and the distribution of photosynthetic products. These vegetation-induced changes can influence the microclimate and nutrient cycling processes within surface soils, thereby regulating microbial community dynamics [72]. Overall, our findings confirm that the structure and diversity of soil microbial communities in the study area are shaped by strong environmental selectivity, mediated through the interplay of soil physicochemical properties, nutrient availability, and vegetation traits [73]. While this study provides important insights into the patterns and drivers of soil microbial community structure and function across different vegetation types in the southern slope of the Qilian Mountains, several limitations should be acknowledged. The sampling was conducted only during a single growing season, which may not fully capture potential temporal variations in microbial community dynamics under seasonal or interannual climatic fluctuations. Moreover, it should be noted that functional assignments from the FUNGuild database may involve some uncertainty, as taxa with lower taxonomic resolution can be associated with more than one, occasionally contrasting, ecological guild. Future studies employing multi-seasonal sampling, broader spatial replication, and functional validation through multi-omics approaches are needed to further elucidate the complex interactions among vegetation, soil, and microbial communities in alpine ecosystems.

## 5. Conclusions

The composition of soil bacterial and fungal phyla remained largely consistent across different vegetation types on the southern slope of the Qilian Mountains. Acidobacteriota and Proteobacteria dominated the bacterial communities, while Basidiomycota and Ascomycota were the principal fungal phyla. Despite this overall compositional similarity, significant variations in microbial diversity, community structure, and functional profiles were observed among vegetation types. Forest soils, characterised by higher moisture content and lower pH, exhibited greater intra-group variability in both bacterial and fungal communities. Fungal diversity was significantly higher in grassland and shrubland soils compared to forests, and bacterial and fungal richness were greater in shrublands than in forests. Chemoheterotrophy was identified as the dominant microbial metabolic function across all vegetation types. However, forest soils displayed enhanced functional potential for carbon and nitrogen cycling relative to grassland and shrubland soils. Notably, shrubland soils, located within ecological transition zones, harbored the highest abundance of microbial functional groups associated with animal parasitism and pathogenicity. Forest soils were predominantly colonized by EcM fungi, largely driven by coniferous stands dominated by *P. crassifolia*, whereas grassland soils exhibited a higher proportion of unclassified fungal functional groups. Soil pH, BD, AN, TP, and L-C were key environmental factors influencing the structure and diversity of bacterial communities, while fungal communities were primarily regulated by pH, BD, L-C, L-N, and SWC. These findings highlight the pivotal role of vegetation types in indirect modulating soil microbial communities through alterations in soil physicochemical conditions. The interactions among vegetation, soil properties, and microbial assemblages are dynamically shaped by spatial environmental heterogeneity in alpine ecosystems.

## Figures and Tables

**Table 1:** Vegetation traits and soil physicochemical and biological parameters under different vegetation types.

Indexes	Grassland	Shrubland	Forest
R-C (g/kg)	353.93 ± 12.58 ^b^	366.71 ± 19.48 ^b^	410.35 ± 6.09 ^a^
R-N (g/kg)	5.55 ± 0.57 ^b^	8.11 ± 0.72 ^a^	6.68 ± 0.74 ^ab^
L-C (g/kg)	364.55 ± 18.55 ^c^	435.28 ± 2.87 ^b^	494.27 ± 4.65 ^a^
L-N (g/kg)	21.22 ± 1.84 ^a^	19.41 ± 1.14 ^a^	12.75 ± 0.72 ^b^
BD (g/cm^3^)	1.33 ± 0.04 ^a^	1.37 ± 0.1 ^a^	1.23 ± 0.16 ^a^
EC (μs/cm)	163.04 ± 32.92 ^a^	170.99 ± 20.62 ^a^	141.4 ± 28.35 ^a^
SWC (%)	4.24 ± 0.49 ^a^	5.51 ± 1.8 ^a^	5.99 ± 1.39 ^a^
Clay (%)	4.21 ± 0.31 ^a^	4.53 ± 0.15 ^a^	3.94 ± 0.59 ^a^
Silt (%)	43.99 ± 2.81 ^a^	45.09 ± 2.29 ^a^	37.68 ± 5.4 ^a^
Sand (%)	51.79 ± 3.11 ^a^	50.38 ± 2.25 ^a^	58.37 ± 5.92 ^a^
SOC (g/kg)	52.13 ± 4.65 ^b^	67.49 ± 9.21 ^ab^	82.59 ± 12.84 ^a^
TN (g/kg)	4.99 ± 0.43 ^a^	6.12 ± 0.76 ^a^	5.98 ± 0.93 ^a^
TP (g/kg)	0.71 ± 0.06 ^a^	0.78 ± 0.04 ^a^	0.63 ± 0.08 ^a^
TK (g/kg)	21.53 ± 0.61 ^a^	20.79 ± 0.98 ^a^	20.14 ± 0.93 ^a^
AN (mg/kg)	336.42 ± 29.9 ^a^	360.12 ± 31.17 ^a^	315.68 ± 32.08 ^a^
AP (mg/kg)	6.09 ± 0.83 ^a^	5.7 ± 0.69 ^a^	7.28 ± 2.7 ^a^
AK (mg/kg)	265.13 ± 38.8 ^a^	261.88 ± 43.46 ^a^	205.75 ± 43.38 ^a^
pH	7.44 ± 0.2 ^a^	7.5 ± 0.15 ^a^	6.78 ± 0.24 ^b^
MBC (mg/kg)	495.51 ± 35.65 ^a^	609.77 ± 73.54 ^a^	715.59 ± 119.16 ^a^
MBN (mg/kg)	37.16 ± 3.14 ^b^	48.05 ± 5.56 ^ab^	59.65 ± 10.16 ^a^
MBP (mg/kg)	14.68 ± 0.72 ^a^	13.7 ± 0.63 ^a^	14.88 ± 0.75 ^a^

Abbreviations: R-C, root carbon concentration; R-N, root nitrogen concentration; L-C, leaf carbon concentration; L-N, leaf nitrogen concentration; BD, bulk density; EC, electrical conductivity; SWC, soil water content; Clay, soil clay fraction; Silt, soil silt fraction; Sand, soil sand fraction; SOC, soil organic carbon; TN, total nitrogen; TP, total phosphorus; TK, total potassium; AN, alkali-hydrolysable nitrogen; AP, available phosphorus; AK, available potassium; MBC, microbial biomass carbon; MBN, microbial biomass nitrogen; and MBP, microbial biomass phosphorus. Data are presented as mean ± standard deviation (SD). The superscript lowercase letters indicate significant differences between vegetation types (*p* < 0.05). Group statistical significance was assessed by one-way ANOVA followed by Duncan’s multiple range test.

## Data Availability

The original contributions presented in this study are included in the article/Supplementary Material. Further inquiries can be directed to the corresponding author.

## Supplementary Materials

The following supporting information can be downloaded at: https://www.mdpi.com/article/10.3390/microorganisms13092075/s1, Table S1: Variance inflation factor (VIF) analysis for environmental factors, and screening out and removing the environmental factors with VIF values greater than 20.
