# Supplementary material for: Impact of Vegetation Type on Taxonomic and Functional Composition of Soil Microbial Communities in the Northeastern Qinghai–Tibet Plateau
Source: Microorganisms. 2025 Sep 6;13(9):2075. doi: 10.3390/microorganisms13092075 (PMC12472663; doi:10.3390/microorganisms13092075)
Supplement: Supplementary file 1 [file microorganisms-13-02075-s001.zip › microorganisms-3729547-supplementary.pdf]

Table S1. Variance inflation factor (VIF) analysis for environmental factors, and screening out and removing the environmental factors with VIF values greater than 20.

Indexes	VIF value	
	**Before screening**	**After screening**
R-C	4.99	2.24
R-N	4.39	3.27
L-C	4.10	2.94
L-N	4.94	4.48
BD	10.71	4.95
EC	7.38	4.72
SWC	14.89	7.34
Clay	14.58	6.81
Silt	7.66	5.12
Sand	20.01	-
SOC	66.94	-
TN	58.33	-
TP	6.20	5.41
TK	12.02	10.33
AN	11.49	8.27
AP	56.88	-
AK	10.79	5.43
pH	6.10	2.94
MBC	108.22	-
MBN	81.85	-
MBP	4.35	2.37

Abbreviations used are as follows: R-C, root carbon concentration; R-N, root nitrogen concentration; L-C, leaf carbon concentration; L-N, leaf nitrogen concentration; BD, bulk density; EC, electrical conductivity; SWC, soil water content; Clay, soil clay fraction; Silt, soil silt fraction; Sand, soil sand fraction; SOC, soil organic carbon; TN, total nitrogen; TP, total phosphorus; TK, total potassium; AN, alkali-hydrolysable nitrogen; AP, available phosphorus; AK, available potassium; MBC, microbial biomass carbon; MBN, microbial biomass nitrogen; and MBP, microbial biomass phosphorus. CD, grassland; GC, shrubland; LD, forest.
